# Effects of two mechanical ventilation strategies during cardiopulmonary bypass on perioperative mechanical power: a prospective observational study

**DOI:** 10.3389/fcvm.2026.1781255

**Published:** 2026-04-30

**Authors:** Mukhsina Akhmadalieva, Gamze Talih, Aydın Tuncay, Isın Gunes, Ayşe Ülgey

**Affiliations:** 1 Department of Anaesthesiology and Reanimation, Erciyes University Medical Faculty, Kayseri, Türkiye; 2 Department of Cardiovascular Surgery, Erciyes University Medical Faculty, Kayseri, Türkiye

**Keywords:** cardiopulmonary bypass, mechanical power, mechanical ventilation, on-pump cardiac surgery, postoperative pulmonary complications

## Abstract

**Background and aim:**

Cardiac surgery requiring cardiopulmonary bypass (CPB) is associated with a high rate of postoperative pulmonary complications (PPCs). Mechanical power (MP) represents energy per breath multiplied by respiratory rate and conversion factor (0.098), resulting in J/min. This prospective observational study aimed to investigate the effects of two ventilation strategies applied during CPB on MP and their association with PPCs.

**Methods:**

In this prospective observational study, ventilation during CPB was managed according to routine clinical practice. Ventilation was discontinued after full CPB flow in Group 1 (*n* = 125), while low-tidal volume ventilation (3 mL/kg) was maintained in Group 2 (*n* = 120). Mechanical power was measured before CPB (T1), after CPB (T2), and in the intensive care unit (T3). Patients were monitored for PPCs for 7 days postoperatively.

**Results:**

MP, the primary endpoint of the study, did not differ significantly between the two ventilation strategy groups at any measured time point. In a secondary exploratory analysis, MP values were higher at T2 and T3 in patients who developed PPCs (T2: 8.54 ± 0.32 vs. 7.78 ± 0.19 J/min, *p* = 0.041; T3: 8.67 ± 0.33 vs. 7.82 ± 0.19 J/min, *p* = 0.029).

**Conclusion:**

The two ventilation strategies applied during CPB did not significantly affect the primary outcome, mechanical power. Higher post-CPB MP values were observed in patients who developed PPCs, although this finding was exploratory.

## Introduction

Cardiovascular diseases are highly prevalent and associated with significant morbidity and mortality (1). On-pump cardiac surgery remains the standard treatment for many cardiac conditions. One of the specificities of cardiac surgery is the use of Cardio-Pulmonary Bypass (CPB). In cardiac surgery with CPB, lung injury is considered almost unavoidable. Contact between blood and the artificial surfaces of the CPB circuit triggers a hyperinflammatory response, which contributes to pulmonary dysfunction. Clinically, lung injury manifests as postoperative pulmonary complications (PPCs), ranging from mild hypoxemia to acute respiratory distress syndrome (ARDS), and occurs in approximately 90% of patients (2).

PPCs following cardiac surgery, such as ventilator associated pneumonia (VAP), atelectasis, and acute respiratory failure, are significant clinical challenges. These complications contribute to increased morbidity and mortality, prolonged intensive care unit (ICU) and hospital stays, and place an additional burden on the healthcare system (3, 4).

The CPB period is particularly critical, as exposure of the patient's blood to artificial surfaces, ischemia-reperfusion injury, and temporary cessation of pulmonary ventilation can all trigger inflammatory responses in lung tissue (5). Recent guidelines on CPB in adult cardiac surgery recommend considering the application of positive end-expiratory pressure (PEEP) and continuing ventilation during CPB as strategies for lung protection (6). However, based on the current evidence, it is unclear as to whether intraoperative use of continuous positive airway pressure (CPAP) or mechanical ventilation during CPB reduces pulmonary complications, the duration of postoperative mechanical ventilation, or the hospital length of stay (7, 8). Although lung protective ventilation strategies are increasingly advocated in cardiac surgery, there is still no consensus regarding the optimal ventilation strategy during CPB (9, 10).

Ventilator-induced lung injury (VILI) results from the interaction between the energy delivered by the ventilator and the lung parenchyma. Mechanical power (MP) reflects the total energy transferred to the lungs during mechanical ventilation. The concept, first introduced by Gattinoni et al. (11), has been proposed as an integrative framework to quantify the energy load applied to the respiratory system and has gained increasing attention in recent years. MP is calculated using multiple ventilatory variables, including tidal volume, respiratory rate, driving pressure, and PEEP, thereby incorporating several determinants traditionally associated with volutrauma, barotrauma, and atelectrauma. Owing to this multidimensional structure, MP may provide a more comprehensive estimate of ventilator load than isolated parameters (12).

However, current evidence, primarily from observational and retrospective studies, indicates that MP is associated with ventilator-related complications and mortality, both during the perioperative period and in postoperative ICU patients (13, 14). In addition, while MP integrates mechanical determinants of lung injury, it does not directly account for biotrauma-related inflammatory processes. Therefore, its role as a predictor of postoperative pulmonary complications remains an area of ongoing investigation.

The primary aim of this study was to evaluate the effect of two different mechanical ventilation strategies applied during CPB on MP in on-pump cardiac surgery. The secondary aim was to explore the association to between MP and PPCs. We hypothesized that maintaining low-tidal-volume ventilation during CPB would influence MP values and may be associated with the incidence of PPCs. The primary outcome of the study was mechanical power, calculated at three predefined perioperative time points.

## Methods

### Patient population

This study was conducted at Erciyes University Faculty of Medicine Hospital following approval from the Erciyes University Clinical Research Ethics Committee (decision number 2023/462). The study was registered at clinicaltrials.gov (NCT06292767).

Adult patients (aged ≥18 years) undergoing elective open-heart surgery under CPB between March 2024 and March 2025 were included in this prospective observational study. All procedures were carried out in accordance with the Declaration of Helsinki, and written informed consent was obtained from all participants. The Strengthening the Reporting of Observational Studies in Epidemiology (STROBE) guidelines were followed in reporting this study (15). The exclusion criteria included emergency cardiac surgery, revision surgery, planned total circulatory arrest, preoperative shock, acute or chronic hypoxemia [partial pressure of arterial oxygen (PaO_2_) < 60 mmHg], mechanical ventilation within 7 days prior to surgery, body mass index (BMI) > 35 kg/m^2^, diagnosis of obstructive sleep apnea syndrome, pulmonary artery pressure >50 mmHg, and a glomerular filtration rate <30 mL/min.

### Study design

After preoperative evaluation, patients were transferred to the operating room. Standard monitoring was applied in all cases, including electrocardiography (ECG), pulse oximetry (SpO_2_), invasive blood pressure, central venous pressure, capnography, urinary catheterization, nasopharyngeal temperature monitoring, the bispectral index (BIS), and near-infrared spectroscopy (NIRS) for cerebral and somatic oxygenation.

General anesthesia was administered following standard monitoring. Anesthesia induction included titrated boluses of propofol (Propofol-PF 1%, Istanbul, Turkey) at 0.5–1 mg/kg. Each patient also received fentanyl 1–2 µg/kg (Talinat® 0.5 mg/10 mL, Vem Pharmaceuticals, Istanbul, Turkey), ketamine 0.5–1 mg/kg (Ketalar 500 mg/10 mL, Pfizer, New York, NY, USA), and rocuronium 1 mg/kg (Esmeron® 50 mg/5 mL, Schering-Plough, the Netherlands) intravenously. After endotracheal intubation, anesthesia was maintained with 1%–2% sevoflurane in a 50/50 mixture of oxygen and air, targeting a BIS of <60. Fentanyl (10 µg/kg/h) and rocuronium (1 mg/kg/h) were administered continuously.

Following intubation, patients were ventilated in the volume-controlled mode using the following settings: a tidal volume of 6–8 mL/kg, a respiratory rate of 10–12 breaths/min, PEEP of 5 cmH_2_O, a fraction of inspired oxygen (FiO_2_) of 0.50, and oxygen saturation maintained above 94% (GE Healthcare Avance™ CS2, GE Healthcare, Chicago, IL, USA). End tidal carbon dioxide (EtCO_2_) was maintained at 35–45 mmHg.

During CPB, ventilation management was determined by the attending cardiac anesthesiologist in cooperation with the surgical team to ensure optimal surgical exposure at full CPB flow. The choice of ventilation strategy was based solely on clinical judgment and routine institutional practice; no allocation was performed as part of a study protocol.

- Group 1 (nonventilated group): Mechanical ventilation was stopped, the anesthesia machine was set to bypass mode, and 150 mL/dk of free-flow air was delivered.
- Group 2 (ventilated group): Patients continued to receive ventilation with a tidal volume of 3 mL/kg, a respiratory rate of 12 breaths/min, PEEP of 5 cmH_2_O, and FiO_2_ of 0.40.

**Figure 1:**
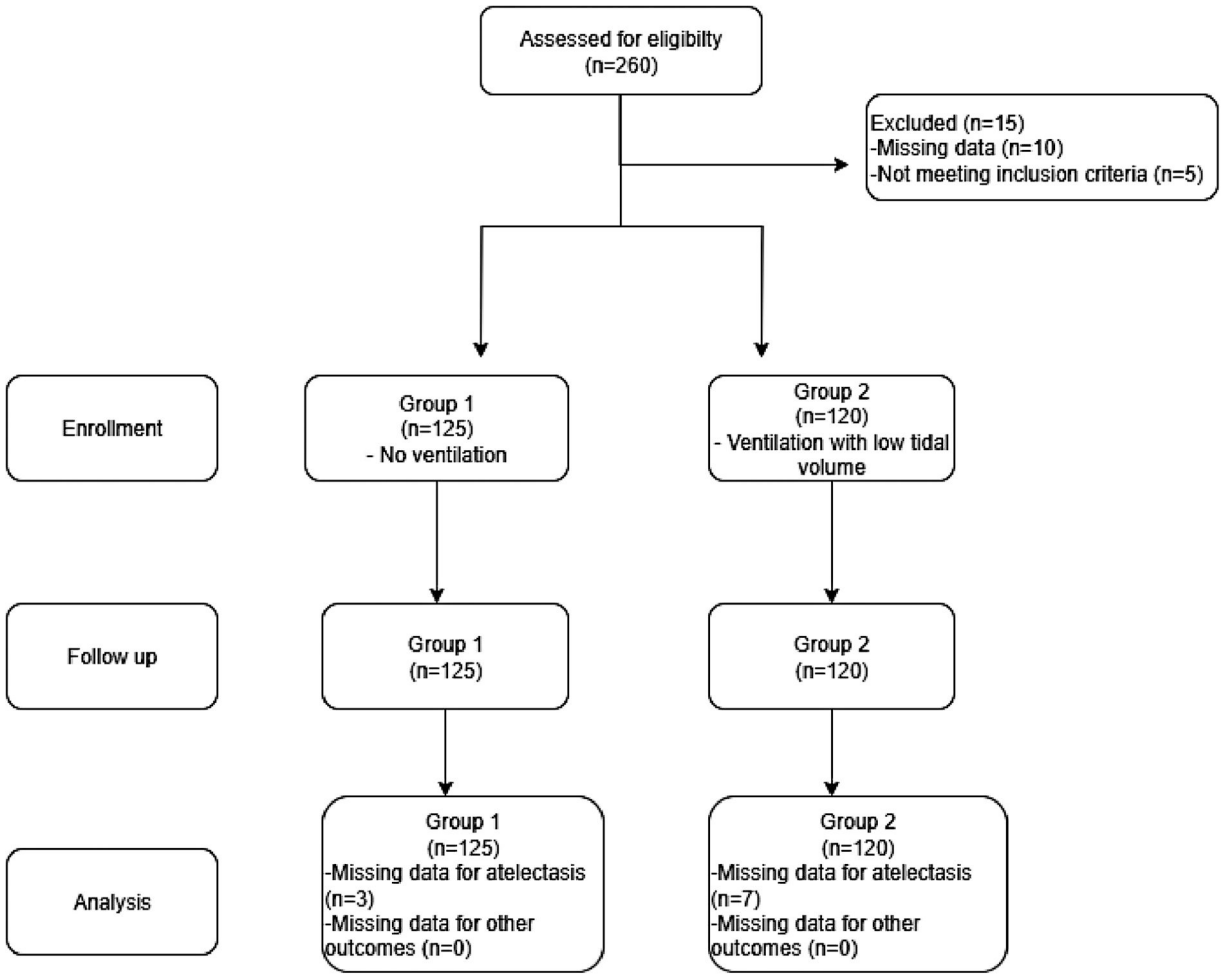
Flow diagram.

At the end of CPB, all patients underwent a single recruitment maneuver at 30 cmH_2_O for 30 s, followed by ventilation with pre-CPB parameters (a tidal volume of 6–8 mL/kg, a respiratory rate of 10–12 breaths/min, EtCO_2_ of 35–45 mmHg, PEEP of 5 cmH_2_O, and FiO_2_ of 0.50) (Figure 1).

The following patient demographics and clinical data were collected: age, height, weight, the American Society of Anesthesiologists (ASA) Physical Status, the surgical procedure, comorbidities, the ejection fraction, smoking history (pack-years), the EUROSCORE II, and the ratio of the forced expiratory volume in 1 s to forced vital capacity (FEV1/FVC).

The following perioperative data were collected: the duration of anesthesia, surgery, and CPB; the aortic cross-clamp time; and transfused blood products (number of units; red blood cells, fresh frozen plasma, platelets, and cryoprecipitate).

The ventilation parameters were recorded at three time points: pre-CPB (T1), post-CPB (T2), and within the first hour in the ICU (T3). These included tidal volume, the respiratory rate, minute ventilation, the peak inspiratory pressure (Ppeak), and the plateau pressure (Pplat). Pplat was measured at end-inspiration during zero flow under volume-controlled ventilation. MP was calculated for each time point using the formula (12):

MP = 0.098 × MV × [Ppeak−0.5 × (Pplat−PEEP)].

The following postoperative data were collected: duration of the ICU stay (h) and hospital stay (days), need for revision surgery, pulmonary complications within 7 days after surgery, and 30-day mortality (presence/absence and day of occurrence).

PPCs were defined as follows: early respiratory failure (PaO_2_/FiO_2_ < 200 within 1 h of ICU transfer), late respiratory failure (requirement for high oxygen support for ≥2 days post-surgery), pneumonia (ventilator-associated or non–ventilator-associated), atelectasis (radiologically confirmed), pneumothorax (radiologically confirmed), and acute respiratory distress syndrome (per Berlin criteria) (16).

Postoperative care was standardized for all patients during the study period. All patients received standardized postoperative analgesia. This included intravenous paracetamol 1 g every 8 h and, if required, intravenous tramadol 50–100 mg. Pain levels were assessed using a numeric rating scale (NRS); additional analgesia was administered for NRS > 4. Patients were maintained in a semi-recumbent position unless contraindicated. Incentive spirometry (Triflo®) and deep-breathing exercises were initiated in the early postoperative period. During intensive care unit follow-up, in-bed mobilization was permitted as clinically appropriate according to the standardized postoperative care protocol.

### Outcomes

The primary outcome of this study was mechanical power (MP), calculated at three predetermined perioperative time points.

Secondary outcomes included the incidence of postoperative pulmonary complications (PPCs) within 7 days after surgery, 30-day all-cause mortality, and the association between MP and the development of PPCs.

## Statistical analysis

Statistical analyses were performed using SPSS Statistics Version 30 (IBM Corp., Armonk, NY, USA). Descriptive statistics are presented as frequency (n) and percentage (%), mean ± standard deviation, or median and interquartile range. The Shapiro–Wilk test was used to assess normality, and Levene's test was used to evaluate homogeneity of variance. A *p*-value < 0.05 was considered statistically significant.

### Sample size calculation

Sample size calculation was based on the primary outcome, namely the effect of two different ventilation strategies on mechanical power. Pilot data from our institution (*n* = 10 per group) were used to estimate the group × time interaction effect, yielding a partial eta squared (*η*²) of 0.0123. This *η*² corresponds to a Cohen's f (effect size) of 0.112, representing a small effect according to Cohen's classification (17). Using this conservative estimate, a two-way repeated-measures ANCOVA design (two groups, three repeated measurements) adjusting for cardiopulmonary bypass (CPB) duration, aortic cross-clamp duration, and surgical type as covariates with *α* = 0.05, power (1–*β*) = 0.95, and an assumed correlation of 0.5 among repeated measures indicated a required total sample size of approximately 210 participants. To account for an anticipated dropout rate of at least 10%, the target enrollment was increased to a minimum of 233 participants.

### Primary outcome analyses

The primary outcome was intraoperative mechanical power (MP) measured at three time points (T1, T2, and T3). Between-group comparisons over time were performed using two-way repeated-measures ANCOVA, including group, time, and group × time interaction effects. The Greenhouse–Geisser correction was applied when sphericity assumptions were violated. Bonferroni correction was used for all pairwise comparisons. Effect sizes (partial eta squared) and 95% confidence intervals were reported to improve interpretability.

### Secondary outcome analyses

Secondary outcomes included postoperative respiratory complications, atelectasis, and other perioperative variables. Continuous variables were compared using independent-samples *t*-test (for normally distributed data) or Mann–Whitney *U* test (for non-normally distributed data). Categorical variables were compared using Pearson's chi-square test, Fisher–Freeman–Halton exact test, Yates' chi-square test, or Fisher's exact test. Effect sizes (Cohen's d for continuous outcomes with 95% confidence intervals) were reported where appropriate.

### Post-hoc and exploratory analyses

When chi-square tests were significant, Bonferroni-adjusted *Z*-tests were used for *post hoc* pairwise comparisons. Additional *post hoc* analyses included exploratory evaluation of the predictive performance of MP at different time points for atelectasis and respiratory complications using receiver operating characteristic (ROC) curve analysis.

## Results

A total of 245 patients were included in the study, with 125 patients in the non-ventilated group and 120 in the ventilated group. The groups did not differ in terms of age, sex, BMI, ASA score, comorbidities, or smoking status (*p* > 0.05). The proportion of coronary surgeries was significantly lower in the non-ventilated group compared with the ventilated group (48% vs. 65%, *p* = 0.007). CPB duration [79.0 (34.0) vs. 97 (51.5) min] and aortic cross-clamp time [46 (27.5) vs. 56 (40) min] were significantly shorter in the non-ventilated group compared with the ventilated group (*p* = 0.001 and 0.006, respectively). The ejection fraction, EuroSCORE II, FEV1/FVC, and anesthesia and surgery durations did not differ between the groups (*p* > 0.05; Table 1).

**Table 1:** Comparison of demographic and intraoperative data between the groups.

Variables	**Group 1**	**Group 2**	Test value	*p*
	*(n* = 125)	*(n* = 120)		
Gender *n* (%)				
Female/Male	50/75 (40/60)	35/85 (29.2/70.8)	3.171	0.075^‡^
Age (year)	59.8 ± 12.8	60.3 ± 12.4	0.261	0.794^†^
BMI (*kg*/*m*^2^)	27.34 ± 4.81	28.05 ± 3.80	1.281	0.201^†^
ASA *n* (%)				
ASA-2/3	1/124 (0.8/99.2)	0/120 (0/100)	-	1.000^ᵵ^
Type of Surgery *n* (%)				
Valve/Coroner	65/60 (52/48)	42/78 (35/65)	7.193	0.007^‡^
Comorbidities *n* (%)			5,495	0,062^Ф^
None	28 (22,4)	15 (12,5)		
Single	40 (32,0)	35 (29,2)		
Multiple	57 (45,6)	70 (58,2)		
Smoke *n* (%)				
+/-	39/86 (31.2/68.8)	43/77 (35.8/64.2)	4.426	0.442^‡^
Ejection Fraction (EF)	55.0 (10.0)	55.0 (10.0)	0.219	0.827^&^
Euro Score	1.04 (1.28)	0.96 (1.11)	1.724	0.085^&^
FEV1/FEVC (%)	77.0 (10.0)	77.0 (6.3)	0.785	0.433^&^
Anesthesia Time (min)	240.0 (70.0)	240.0 (60.0)	1.246	0.213^&^
Surgery Duration (min)	210.0 (60.0)	210.0 (63.8)	0.883	0.377^&^
CPB Time (min)	97.0 (51.5)	79.0 (34.0)	3.253	0.001^&^
Cross-Clamp Time (min)	56.0 (40.0)	46.0 (26.5)	2.752	0.006^&^

Group 1 = no ventilation during CPB; Group 2 = ventilation with 3 mL/kg during CPB. Statistical analysis.

† Independent sample *t* test.

& Mann–Whitney *U* test.

‡ Pearson chi- square test.

Ф Yates chi-square test.

ᵵ Fisher exact test, mean ± SD; median (IQR) *p* < 0.05 significantly.

Table 2 presents the airway pressures, minute ventilation, and MP for both groups. At each time point, MP did not differ within or between groups (*p* > 0.05). The T3 Pplat was significantly higher in the ventilated group compared with the non-ventilated group (*p* = 0.015). Intra-group comparisons revealed significant differences in Pplat, Ppeak, and minute ventilation (*p* < 0.05; Table 2).

**Table 2:** Comparison of airway pressure and mechanical power values at T1, T2 and T3 periods between groups.

Variables	**Group 1**	**Group2**	Test value	* p*
	*(n* = 125)	*(n* = 120)		
Plateau pressure (cmH_2_0)				
T1	13.27 (7.57–18.97)*^a^*	13.07 (6.66–19.48)*^a^*	0.269	0.604
T2	13.45 (7.53–19.37)*^a^*	13.61 (7.48–19.74)*^ab^*	0.160	0.690
T3	14.85 (9.36–20.34)*^b^*	13.98 (8.57–19.39)*^b^*	6.047	0.015
^γ^: *F*; *p*	20.699; <0.001	5.589; 0.004		
GroupXTime interaction effect: *F* = 4.097; *p* = 0.018; *partial eta squared*=0.033; *powe*r=0.723				
Peak pressure (cmH_2_0)				
T1	19.07 (11.27–26.87)*^a^*	18.77 (10.93–26.61)*^ab^*	0.359	0.550
T2	19.00 (12.08–25.92)*^a^*	19.16 (11.42–26.90)*^a^*	0.109	0.741
T3	18.01 (11.27–24.75)*^b^*	18.03 (11.60–24.46)*^b^*	0.003	0.953
^γ^: *F*; *p*	6.357; 0.002	6.232; 0.002		
GroupxTime interaction effect: *F* = 1.031; *p* = 0.358; *partial eta squared*=0.009; *powe*r=0.229				
Minute ventilation (MV) (L/min)				
T1	5.44 (2.91–7.97)*^a^*	5.70 (3.84–7.56)*^a^*	4.834	0.070
T2	5.38 (3.30–7.46)*^a^*	5.49 (3.26–7.72)*^b^*	0.673	0.413
T3	6.08 (3.38–8.78)*^b^*	6.22 (3.61–8.83)*^c^*	0.633	0.427
^γ^: *F*; *p*	15.037; <0.001	15.825; <0.001		
GroupxTime interaction effect: *F* = 0.783; *p* = 0.458; *partial eta squared*=0.007; *powe*r=0.183				
Mechanical Power (MP) (J/min) (adjusted for CPB duration, cross-clamp duration, surgical types)**				
T1	8.29 (7.72–8.86)	8.23 (7.68–8.78)	0.024	0.878
T2	7.91 (7.44–8.38)	8.06 (7.61–8.51)	0.164	0.686
T3	7.89 (7.42–8.36)	8.21 (7.74–8.68)	0.916	0.339
^γ^**:** *F*; *p*	1.122; 0.327	0.310; 0.734		
GroupxTime interaction effect: *F* = 0.264; *p* = 0.768; *partial eta squared* = 0.002; *powe*r = 0.091				

Group 1 = no ventilation during CPB; Group 2 = ventilation with 3 mL/kg during CPB. Mean (95% CI).

γ Comparisons between measurement time points within each group, F: Two-way analysis of variance for repeated measures.

** Two-way analysis of covariance for repeated measures Superscripts a, b, and c indicate significant differences between measurement time points within each group. Same superscript do not differ significantly. *p* < 0.05 significantly.

Blood product transfusions, the hospital length of stay, and the reoperation rate did not differ between the groups (*p* > 0.05). However, the ICU length of stay was significantly shorter in the ventilated group compared with the non-ventilated group (*p* < 0.001). The incidence of atelectasis was 23.8% in the non-ventilated group and 23.0% in the ventilated group, with no difference between the groups (*p* > 0.05). There were also no significant differences between the groups in terms of PPCs and mortality (*p* > 0.05; Table 3).

**Table 3:** Comparison of postoperative complication rates and length of hospital stay between groups.

Variables	Group 1	Group 2	Test value	*p*
	*(n* = 125)	*(n* = 120)		
Tranfused of blood products (Number of units)	2.0 (2.0)	1.5 (2.3)	0.124	0.901^&^
Length of ICU stay (hours)	48.0 (24.0)	32.0 (21.0)	4.224	<0.001^&^
Length of hospital stay (days)	7.0 (3.0)	7.0 (2.8)	0.593	0.553^&^
Revision surgery, *n* (%)				
Yes/No	5/120 (4.0/96.0)	5/115 (4.2/95.8)	-	1.000^ᵵ^
Atelectasis, *n* (%)	*(n* = 122)	*(n* = 113)		
Yes/No	29/93 (23.8/76.2)	26/87 (23.0/77.0)	0.019	0.890^Ф^
Pulmoner complication, *n* (%)				
Yes/No	32/93 (25.6/74.4)	33/87 (27.5/72.5)	0.113	0.736^‡^
30-day mortality, *n* (%)				
Yes/No	9/116 (7.2/92.8)	9/111 (7.5/92.5)	0.001	0.999^Ф^
Time to death (days)	4.0 (8.0)	6.0 (11.0)	0.492	0.623^&^

Group 1 = no ventilation during CPB; Group 2 = ventilation with 3 mL/kg during CPB. Statistical analysis:.

& Mann–Whitney *U* test.

‡ Pearson kikare testi.

Ф Yates kikare testi.

ᵵ Fisher exact test, median (IQR) ***p*** < 0.05 significantly.

**Figure 2:**
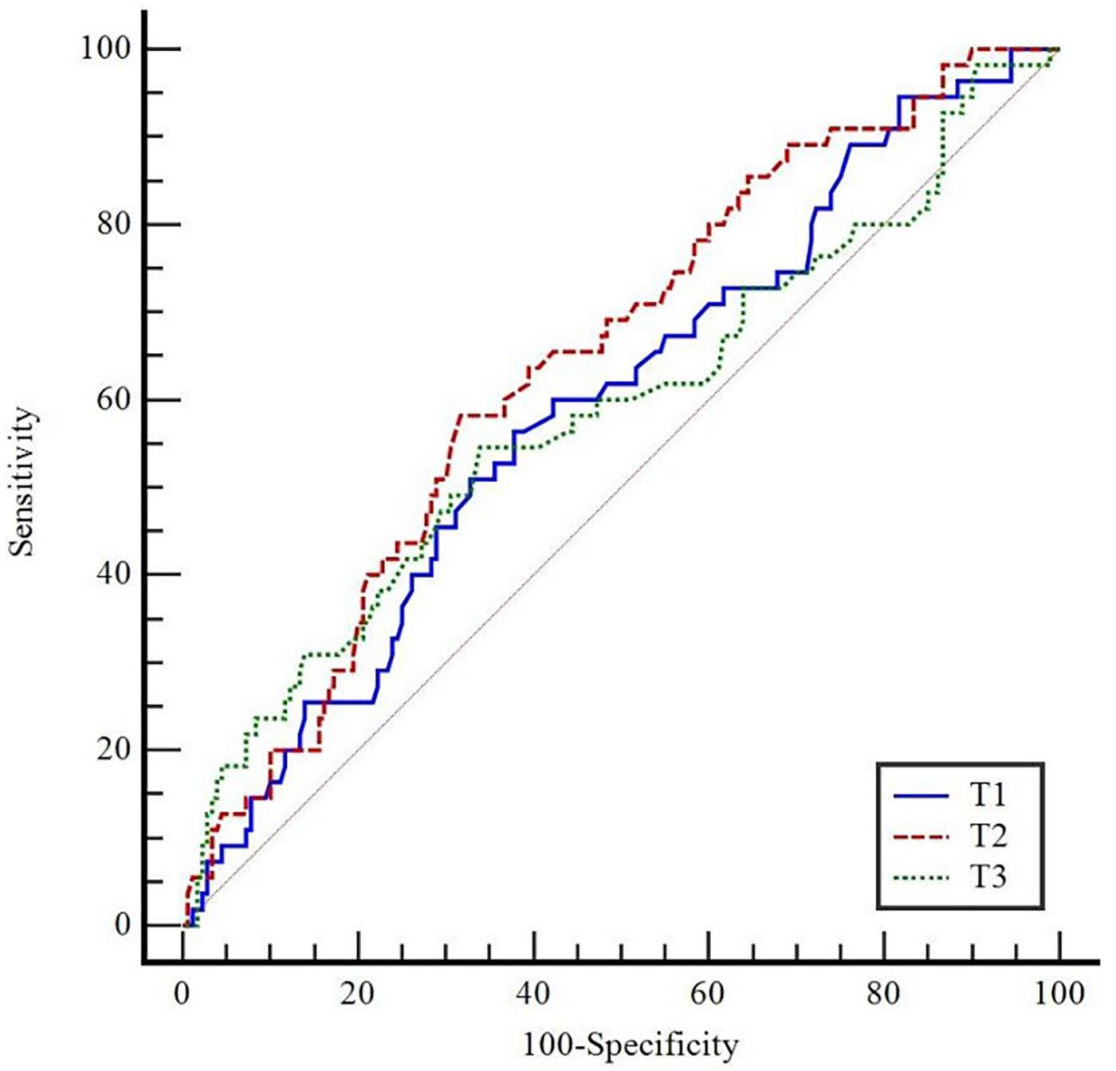
ROC curves of mechanical power (MP) values at T1, T2, and T3 for predicting atelectasis.

Patients who developed atelectasis had a significantly higher MP compared with patients who did not develop atelectasis at T2 (*p* < 0.001; Table 4). ROC curve analysis was used to evaluate the predictive performance of MP at T1, T2, and T3 for atelectasis (Figure 2). The analysis showed that MP at T1 (*p* = 0.032) and T2 (*p* < 0.001) had statistically significant predictive performance, whereas MP at T3 did not reach statistical significance (*p* > 0.05; Table 5).

**Table 4:** Comparison of mechanical power (MP) values according to the presence of atelectasis (adjusted for CPB duration, cross-clamp duration, surgical types).

Variables	Atelectasis		Tests*^δ^*	
	No	Yes	Test value	*p*
	*(n* = 180)	*(n* = 55)		
MP (J/min)				
T1	8.15 (7.76–8.54)	8.27 (7.86–8.68)	5.098	0.085
T2	7.87 (7.56–8.18)	8.22 (7.89–8.55)	20.375	<0.001
T3	8.00 (7.67–8.33)	8.13 (7.46–8.80)	2.596	0.108
^γ^: *F*; *p*	1.234; 0.293	0.521; 0.595		
GroupxTime interaction effect: *F* = 1.099; *p* = 0.335; *partial eta squared*=0.010; *powe*r=0.242				

Mean (95% CI).

^δ^ Comparisons between groups at each measurement time point.

γ Comparisons between measurement time points within each group, *F*: Two-way analysis of covariance for repeated measures, *p* < 0.05 significantly.

**Table 5:** Predictive value of mechanical power (MP) in diagnosing atelectasis.

Variables	*AUC*	*p*	Cut-off	Sensitivity (%)	Specificity (%)	PPV	NPV
	(%95 GA)			(95% CI)	(95% CI)	(95% CI)	(95% CI)
T1	0.593 (0.527–0.656)	0.032	>7.99	56.4 (42.3–69.7)	62.2 (54.7–69.3)	31.3 (25.3–38,1)	82,4 (77.2–86.5)
T2	0.644 (0.579–0.705)	<0.001	>8.45	58.2 (44.1–71.3)	68.3 (61.0–75.1)	36.0 (29.2–43.4)	84.2 (79.4–88.1)
T3	0.587 (0.521–0.651)	0.063	>8.2	54.6 (40.6–68.0)	66.1 (58.7–73.0)	33.0 (26.4–40.3)	82.6 (77.8–86.6)

AUC, area under the curve; CI, confidence interval; Cut-off, optimal threshold value; PPV, positive predictive value; NPV, negative predictive value; *p* < 0.05 significantly.

**Figure 3:**
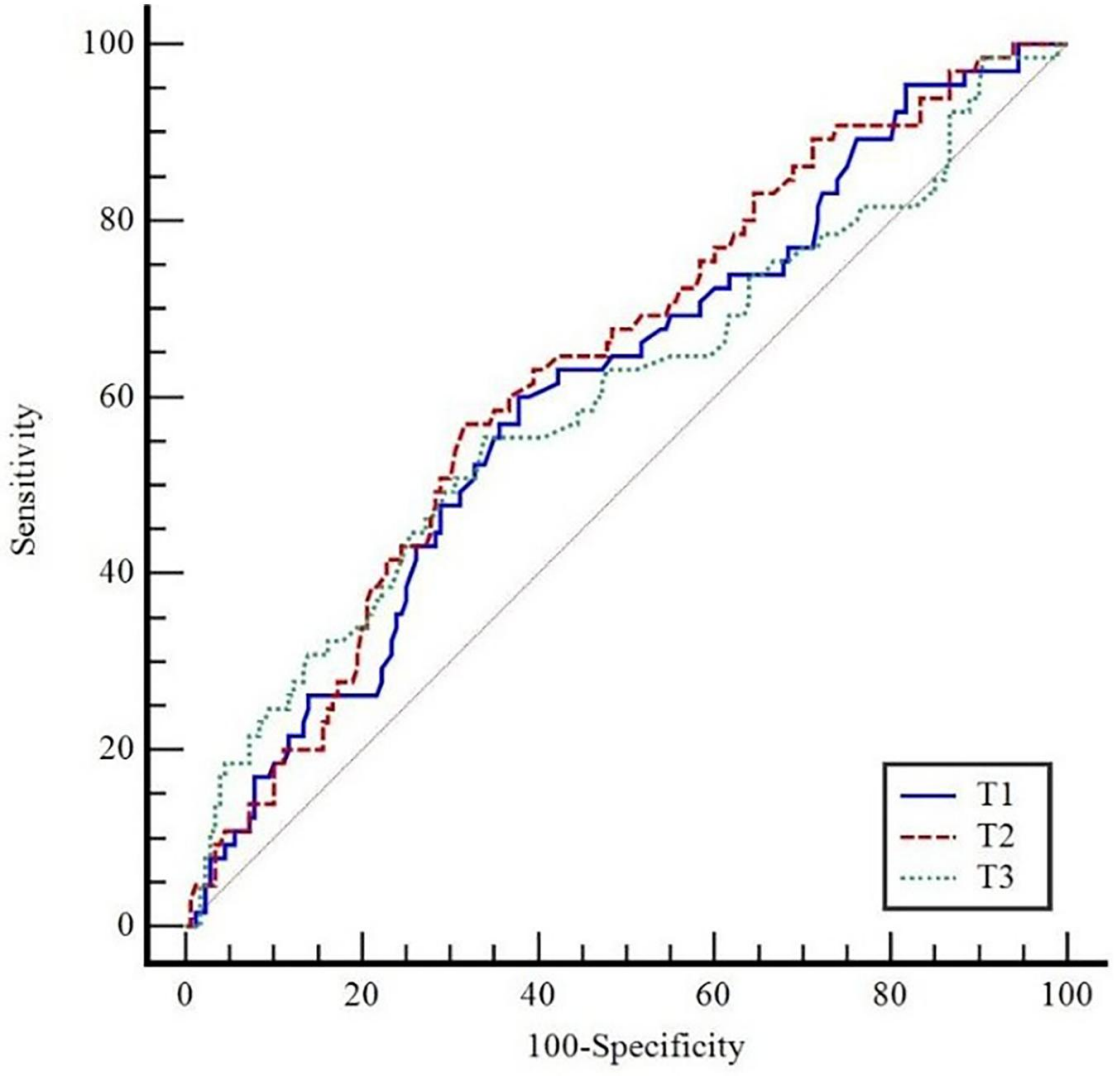
ROC curves of mechanical power (MP) values at T1, T2, and T3 for predicting postoperative pulmonary complications.

 As shown in Table 6, patients with PPCs had a significantly higher MP compared with patients without PPCs at T2 (*p* = 0.041), T3 (*p* = 0.029). The predictive value of MP at T1, T2, and T3 for PPCs was also assessed using ROC curve analysis (Figure 3). MP at T1 (*p* = 0.007), T2 (*p* = 0.001), and T3 (*p* = 0.021) all demonstrated statistically significant predictive performance (Table 7).

**Table 6:** Comparison of mechanical power (MP) values according to postoperative pulmonary complications (PPC). (adjusted for CPB duration, cross-clamp duration, surgical types).

Variables	PPC		Tests*^δ^*	
	No	Yes	Test value	*p*
	*(n* = 180)	*(n* = 65)		
MP (J/min)				
T1	8.06 (7.61–8.51)	8.67 (7.91–9.43)	1.792	0.182
T2	7.78 (7.41–8.15)	8.54 (7.91–9.17)	4.224	0.041
T3	7.82 (7.45–8.19)	8.67 (8.02–9.32)	4.832	0.029
^γ^: *F*; *p*	0.914; 0.402	0.100; 0.905		
GroupxTime interaction effect: *F* = 0.304; *p* = 0.738; *partial eta squared*=0.003; *powe*r=0.098				

Mean (95% CI).

^δ^ Comparisons between groups at each measurement time point.

γ Comparisons between measurement time points within each group, *F*: Two-way analysis of covariance for repeated measures, *p* < 0.05 significantly.

**Table 7:** Predictive value of mechanical power (MP) for postoperative pulmonary complications (PPC).

Variables	*AUC*	*p*	Cut-off	Sensitivity (%)	Specificity (%)	PPV	NPV
	(%95 CI)			(%95 CI)	(%95 CI)	(%95 CI)	(%95 CI)
T1	0.609 (0.544–0.670)	0.007	>7.99	60.0 (47.1–72.0)	62.2 (54.7–69.3)	36.4 (30.4–43.0)	81.2 (75.8–85.6)
T2	0.634 (0.570–0.694)	0.001	>8.45	56.9 (44.0–69.2)	68.3 (61.0–75.1)	39.4 (32.4–46.7)	81.5 (76.6–85.5)
T3	0.600 (0.535–0.662)	0.021	>8.2	55.4 (42.5–67.7)	66.1 (58.7–73.0)	37.1 (30.4–44.3)	80.4 (75.4–84.6)

AUC, area under the curve; CI, confidence interval; Cut-off, optimal threshold value; PPV, positive predictive value; NPV, negative predictive value; *p* < 0.05 significantly.

## Discussion

In this prospective observational study, intraoperative MP values did not differ between ventilation strategies, and no significant difference in PPC incidence was observed between groups. Nevertheless, patients who developed PPCs exhibited higher MP values in the early post-CPB period. These results highlight MP as a potential associative marker, but the study's design and limitations preclude any causal or predictive conclusions.

Several patient- and procedure-related factors contribute to the development of PPCs after cardiac surgery, including obesity, smoking, a systemic inflammatory response during CPB, hyperoxia, and prolonged postoperative mechanical ventilation (18). Differences in patient populations and PPC definitions in previous studies have resulted in reported incidences ranging from 25% to 50% (9, 19, 20). In the study by Becker et al. (2), when mild hypoxemia was included, the incidence of PPCs increased to approximately 90%. In the current study, the PPC rate was 26.5%, lower than previously reported. This may be attributed to shorter CPB and cross-clamp durations, a lower prevalence of existing pulmonary disease, reduced transfusion requirements, and the exclusion of complex surgeries. Standardizing the postoperative pain protocol and implementing respiratory exercises (e.g., early use of spirometry and mobilization) may have also played a role in reducing PPCs.

Ventilation during CPB is a critical component of intraoperative mechanical ventilation management and is typically determined by anesthesiologists in the operating room. Adjustable parameters in ventilation strategies include CPAP, low tidal volume ventilation, PEEP, and vital capacity maneuvers (VCMs). Existing research on whether ventilation during CPB improves respiratory outcomes remains controversial. Nevertheless, it is well established that a high tidal volume is associated with worse clinical outcomes (21). A lower driving pressure (<16 cmH_2_O), a tidal volume below 8 mL/kg/min, and the use of recruitment maneuvers have all been linked to a reduction in PPCs (3). Lung-protective mechanical ventilation strategies (with a tidal volume of 6–8 mL/kg) are fundamental in preventing PPCs. In a randomized controlled trial by Becker et al. (2), perioperative MP values were lower in patients ventilated with flow-controlled modes compared with pressure-controlled ventilation. This reduction was mainly due to lower respiratory rates rather than tidal volume, and was associated with a lower incidence of PPCs, suggesting that, exploratorily, targeting lower MP may improve postoperative pulmonary outcomes. In this study, all patients were ventilated with a tidal volume of 6–8 mL/kg tidal volumes and PEEP of 5 cmH_2_O before and after CPB, with respiratory rates standardized across all patients, which may have contributed to the relatively low incidence of PPCs.

Studies have shown that maintaining mechanical ventilation or positive airway pressure during CPB improves early postoperative gas exchange and reduces the inflammatory response (22). In the MECANO trial, intraoperative ventilation with a low tidal volume during CPB in cardiac surgery did not reduce mortality, early respiratory failure, the need for ventilation beyond postoperative day 2, or reintubation, compared with patients who were not ventilated. However, there was a benefit in the subgroup undergoing isolated coronary surgery (10). Similarly, the PROVECS trial showed that high PEEP and recruitment maneuvers did not significantly improve postoperative outcomes and may even cause lung over distension (9). In their observational study, Trancart et al. (23) reported no significant differences in the end-expiratory lung volume, the PaO_2_/FiO_2_ ratio, ventilation duration, or the frequency of atelectasis between ventilated and non-ventilated groups during CPB. These findings suggest that insisting on maintaining ventilation during CPB may not be sufficient on its own to reduce PPCs, especially considering the difficulty of preserving a comfortable surgical work.

Most of the literature on MP comes from ICU patients and noncardiac surgery, while data on cardiac surgery remain relatively limited. The present study contributes to this growing body of evidence by evaluating the relationship between intraoperative MP and postoperative pulmonary complications (PPCs) in patients undergoing open-heart surgery with an arrested heart. MP integrates major lung injury mechanisms—volutrauma, barotrauma, atelectrauma—into a single parameter, and is regarded as a comprehensive indicator of VILI (24). The type of surgery, patient positioning, and body habitus can directly influence MP. Studies in robotic laparoscopic surgeries have shown that Trendelenburg positioning, pneumoperitoneum, and obesity significantly increase MP and are associated with the development of atelectasis (25). During thoracic surgery, lateral positioning and one-lung ventilation have been reported to increase MP and lung elastance, thereby increasing the risk of complications (26). Although these previous prospective studies have reported an association between lower perioperative MP and reduced rates of certain PPCs, these findings were based on secondary, hypothesis-generating outcomes and should therefore be interpreted with caution. Within this context, our results add further observational evidence supporting a potential association between intraoperative MP and PPCs.

Several formulas have been proposed to calculate mechanical power (MP). The classical equation incorporates plateau pressure (Pplat), which is typically measured at end-inspiration during volume-controlled ventilation (11, 12). Alternative simplified equations that do not require Pplat have also been described; however, these may be associated with a slight reduction in accuracy (27). In the present study, MP was calculated using the bedside equation described by Chiumello et al. (12), based on routinely recorded ventilator parameters including Ppeak, Pplat, PEEP, and minute ventilation.

No universally accepted cutoff for MP has been established. Values in the range of 4–7 J/min have been associated with minimal lung injury when assessed using anatomical and physiological variables (28). These findings suggest that MP may not serve as a highly accurate standalone predictor, but rather as a complementary tool for early identification of patients at risk for complications. Additionally, the high high negative predictive value (84.2%) of MP indicates that it may be especially useful in ruling out risk in low-risk patients. MP appears to offer statistically significant yet modest predictive value for postoperative complications and may serve as a supplementary risk marker in clinical decision-making.

A potential limitation of this study is that the ventilation strategy during CPB was determined based on clinical judgment rather than randomized allocation. However, all consecutive eligible patients were included, and no exclusions were made based on anticipated intraoperative conditions, which minimizes the risk of systematic selection bias. In addition, there were baseline imbalances between the groups, and we did not perform formal adjustment for potential confounders. The analyses of postoperative pulmonary complications (PPCs) were exploratory in nature and should be interpreted with caution. Finally, the single-center design and limited sample size may restrict the generalizability of our findings. Given the multifactorial nature of PPCs, further multicenter studies with larger cohorts are needed to evaluate additional contributing factors beyond mechanical power.

## Conclusion

MP measured at different intraoperative time points in on-pump cardiac surgery was found to be associated with PPCs in exploratory analyses. However, the two ventilation strategies used during CPB did not significantly affect MP in this patient population. The role of MP in guiding dynamic and individualized ventilation strategies is gaining increasing importance.

## Data Availability

The original contributions presented in the study are included in the article/Supplementary Material, further inquiries can be directed to the corresponding author.
